# Fetal growth restriction in a genetic model of sporadic Beckwith–Wiedemann syndrome

**DOI:** 10.1242/dmm.035832

**Published:** 2018-11-16

**Authors:** Simon J. Tunster, Mathew Van de Pette, Hugo D. J. Creeth, Louis Lefebvre, Rosalind M. John

**Affiliations:** 1 Cardiff School of Biosciences, Cardiff University, Cardiff CF10 3AX, UK; 2 Department of Medical Genetics, Life Sciences Institute, University of British Columbia, Vancouver, BC V6T 1Z3, Canada

**Keywords:** Beckwith–Wiedemann syndrome, Mouse model, Fetal growth restriction, Placentomegaly

## Abstract

Beckwith–Wiedemann syndrome (BWS) is a complex imprinting disorder involving fetal overgrowth and placentomegaly, and is associated with a variety of genetic and epigenetic mutations affecting the expression of imprinted genes on human chromosome 11p15.5. Most BWS cases are linked to loss of methylation at the imprint control region 2 (ICR2) within this domain, which in mice regulates the silencing of several maternally expressed imprinted genes. Modelling this disorder in mice is confounded by the unique embryonic requirement for *Ascl2*, which is imprinted in mice but not in humans. To overcome this issue, we generated a novel model combining a truncation of distal chromosome 7 allele (DelTel7) with transgenic rescue of *Ascl2* expression. This novel model recapitulated placentomegaly associated with BWS, but did not lead to fetal overgrowth.

## Abstract (precis)

**Summary:** A novel genetic mouse model of sporadic Beckwith–Wiedemann syndrome (BWS) recapitulates placentomegaly, but placental defects lead to late gestation fetal growth restriction, which contrasts with the fetal overgrowth characteristic of BWS in humans.

## INTRODUCTION

Beckwith–Wiedemann syndrome (BWS; MIM #130650) is a complex imprinting disorder associated with a range of growth and developmental phenotypes, including overgrowth, macroglossia, abdominal wall defects and an increased frequency of childhood tumours (Brioude et al., 2018; Lapunzina, 2005; Weksberg et al., 2010). Historically, BWS has been diagnosed through the presence of three or more ‘major’ criteria, including abdominal wall defects, macroglossia (enlarged tongue), macrosomia (birth weight >97th percentile), ear creases/pits and visceromegaly (enlarged abdominal organs). BWS may also be diagnosed on presentation with two major criteria and at least one ‘minor’ criterion, including placental defects, placentomegaly, neonatal hypoglycaemia and cardiomegaly (Õunap, 2016; Weksberg et al., 2010). However, a recent Consensus Statement proposes a re-evaluation of diagnostic criteria, describing several cardinal features (including macroglossia, lateralised overgrowth and placental mesenchymal dysplasia) alongside additional suggestive features (including placentomegaly and fetal overgrowth) (Brioude et al., 2018). The prevalence of BWS is estimated at 1 in 13,700 live births (Thorburn et al., 1970). The majority of cases occur sporadically, while ∼15% are inherited, for instance via loss of a functional maternal *CDKN1C* allele (Weksberg et al., 2010, 2005, 2003).

BWS is caused by genetic or epigenetic mutations that disrupt the expression of one or more imprinted genes, which, unlike most autosomal genes, are expressed predominantly from one parental allele (Ferguson-Smith and Surani, 2001). The parent-of-origin-specific expression of imprinted genes is regulated through mechanisms that include differential methylation and expression of long noncoding RNAs (Delaval and Feil, 2004; Koerner et al., 2009; O'Neill, 2005). Over 100 imprinted genes have been identified in mice, with around half of these known to be also imprinted in humans (Ishida and Moore, 2013). Most imprinted genes have critical roles in regulating fetal and/or placental growth (Cleaton et al., 2014; Tunster et al., 2013), with the parental conflict hypothesis predicting that paternally expressed imprinted genes promote growth and maternally expressed imprinted genes restrict growth (Moore and Haig, 1991).

BWS results from genetic or epigenetic defects within a ∼1 Mb imprinted region of human chromosome 11p15.5 (Koufos et al., 1989; Ping et al., 1989). Imprinting of genes within this domain is associated with differential methylation of two imprinting control regions: ICR1 and ICR2 (also known as IC1 and IC2) (Du et al., 2003, 2004). Methylation of the paternal ICR1 suppresses the noncoding *H19* RNA whilst permitting expression of the growth-enhancing *IGF2*. On the maternal chromosome, absence of ICR1 DNA methylation is associated with expression of *H19* and suppression of *IGF2* (Ideraabdullah et al., 2008). In contrast, maternal methylation of ICR2 prevents transcription of the long noncoding RNA *KCNQ1OT1*, which is predicted to permit the expression of several genes, including the growth-limiting *CDKN1C* and *PHLDA2*. On the paternal chromosome, absence of ICR2 methylation permits transcription of *KCNQ1OT1* and is associated with silencing in *cis* of the imprinted genes in the region (Du et al., 2004). Several genetic and epigenetic defects have been associated with BWS, including paternal uniparental disomy (pUPD), gain of ICR1 methylation, loss of ICR2 methylation and mutations within the *CDKN1C* coding region (Weksberg et al., 2010, 2005, 2003). Whereas maternally inherited *CDKN1C* mutations account for nearly half of familial cases, only ∼5% of sporadic cases are associated with such mutations (Lam et al., 1999; Lee et al., 1997). Instead, the majority of sporadic cases are attributable to loss of ICR2 methylation, which effectively silences the expression of several maternally expressed genes regulated by *KCNQ1OT1*, including *CDKN1C* and *PHLDA2* (Gaston et al., 2001; Lee et al., 1999; Smilinich et al., 1999).

Human 11p15.5 is syntenic with mouse distal chromosome 7, with the exception that, in humans, ICR1 is located towards the telomere and ICR2 towards the centromere, whereas in the mouse this orientation is reversed. Despite conservation of the BWS region between species, modelling BWS in mice remains a complex undertaking. Loss of function of *CDKN1C*, seen most often in familial BWS, has been modelled by the generation of three independent *Cdkn1c* mutant alleles, which recapitulate some aspects of the disorder (Takahashi et al., 2000; Yan et al., 1997; Zhang et al., 1997). However, these models are of limited relevance as BWS is typically sporadic and predominantly associated with loss of methylation at ICR2, which is predicted to lead to the silencing of all the maternally expressed genes within the domain.

**Fig. 1.**
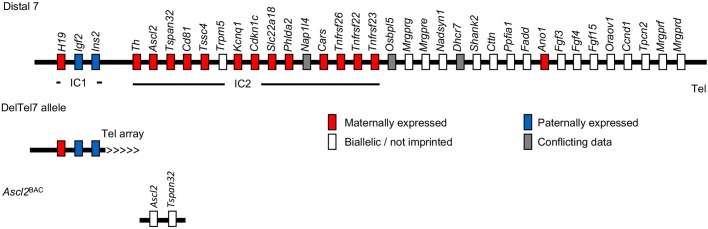
**Schematic of DelTel7 and *Ascl2*^BAC^ models.** Schematic of mouse distal chromosome 7 showing the extent of the DelTel7 deletion, which encompasses the entire IC2 domain and ∼20 telomeric genes, whilst leaving the IC1 domain intact. Imprinting status from Tunster et al. (2013).

The generation of a mouse model possessing a truncation of distal chromosome 7 (DelTel7) provided an important first step towards creating a mechanistic model of loss of expression of imprinted genes associated with loss of ICR2 methylation in human (Oh et al., 2008). The DelTel7 truncation encompasses the entire IC2 imprinted domain and an additional ∼20 nonimprinted genes located at the telomeric end of mouse distal chromosome 7, but with imprinted expression of the IC1 maintained (Fig. 1). Paternal inheritance of the ∼2.6 Mb DelTel7 deletion allele caused no adverse phenotype, consistent with the paternally silenced status of protein-coding genes within the domain as well as absence of haploinsufficiency effects. However, maternal inheritance of the deletion resulted in embryonic lethality by embryonic day (E) 10.5 (Oh et al., 2008). Placentae of maternal DelTel7 heterozygotes were characterised by loss of the junctional zone and an expanded giant cell layer, a phenotype reminiscent of *Ascl2* loss of function (Guillemot et al., 1994; Oh et al., 2008). Indeed, reactivation of the paternal IC2 domain by paternal transmission of an IC2 knockout (KO) allele (KvDMR1 KO) was sufficient to rescue embryonic lethality and restore placental structure, thus attributing the lethality and placental defects to loss of expression of one or more genes within the IC2 domain (Oh-McGinnis et al., 2010).

A key difference in imprinting between human and mouse in the BWS region is the status of *ASCL2*/*Ascl2*, which in mice is maternally expressed, but escapes imprinting in humans (Miyamoto et al., 2002; Monk et al., 2006). The biallelic expression of *ASCL2* in humans might explain the survival of human conceptuses with ICR2 hypomethylation, with *ASCL2* expression maintained from the active paternal allele, whereas loss of IC2 imprinting in the mouse completely ablates *Ascl2* expression. We recently reported the generation of a transgenic mouse carrying a BAC transgene spanning the *Ascl2* gene (*Ascl2*^BAC^) (Reed et al., 2012; Tunster et al., 2016). Here, we asked whether transgenic *Ascl2* expression could rescue the embryonic lethality in the context of maternal DelTel7 inheritance, to allow a later characterisation of this model.

## RESULTS

### Restoring *Ascl2* expression rescues DelTel7 lethality

We first explored whether restoring *Ascl2* gene expression could rescue the embryonic lethality associated with deletion of the maternal IC2 domain by mating hemizygous males carrying *Ascl2*^BAC^ with heterozygous females that inherited the DelTel7 allele from their father. Full rescue should result in viability beyond E10.5 of three genotypes in equal proportions: fully wild-type (WT) embryos inheriting neither genetic alteration; *Ascl2*^BAC^ embryos inheriting only the transgene from their father; and DelTel7; *Ascl2*^BAC^ double mutants (called DelTel7^BAC^) inheriting the DelTel7 allele from their mother and *Ascl2*^BAC^ from their father. DelTel7 embryos inheriting only the deletion allele should not be observed beyond ∼E10.5 (Oh et al., 2008). Twenty-two litters were analysed at E14.5, comprising 158 viable conceptuses, of which 35 were DelTel7^BAC^ (Table 1); and 26 litters were analysed at E18.5, comprising 147 viable conceptuses, of which 30 were DelTel7^BAC^ (Table 1). The presence of the transgene successfully, although not completely, rescued lethality imposed by loss of function of the maternally expressed genes within the IC2 domain, allowing a phenotypic assessment of the rescued conceptuses.

**Table 1.**
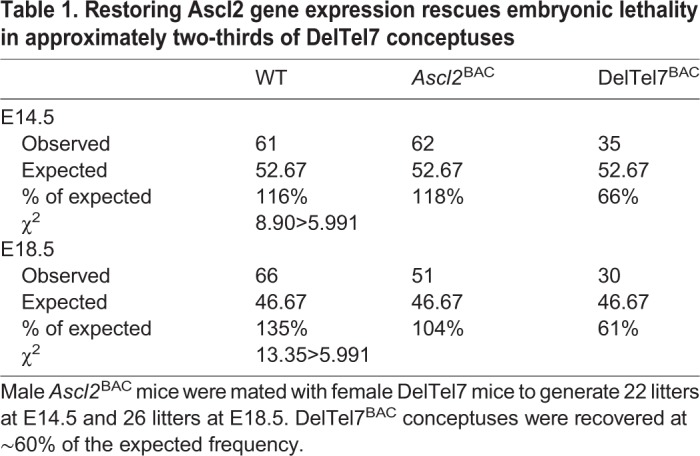
**Restoring Ascl2 gene expression rescues embryonic lethality in approximately two-thirds of DelTel7 conceptuses**

### Placentomegaly and fetal growth restriction of DelTel7^BAC^ conceptuses

**Fig. 2.**
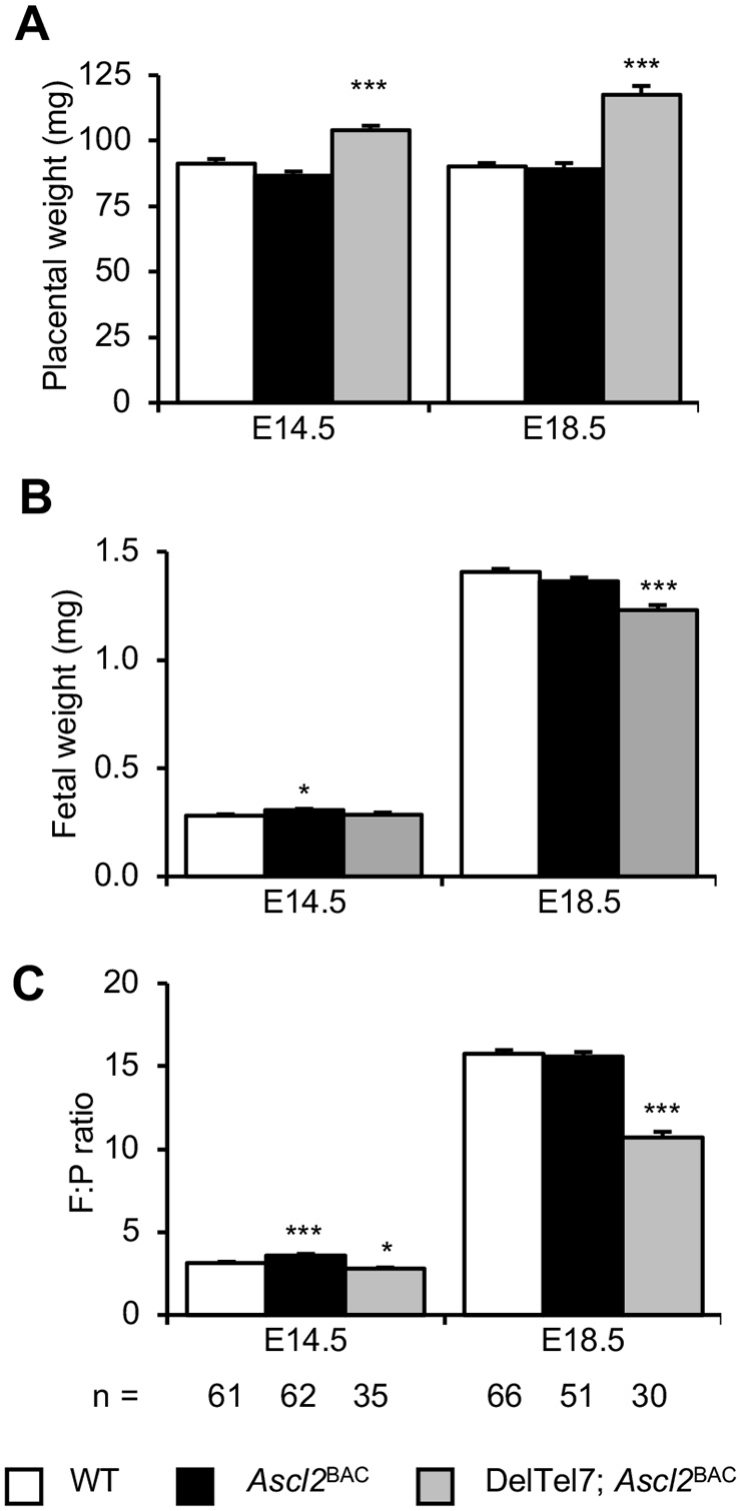
**Restoring *Ascl2* gene function rescues DelTel7 lethality.** (A) Placental wet weights at E14.5 and E18.5 for the three viable genotypes generated from mating *Ascl2*^BAC^ (129) males with DelTel7 (CD1) females. *Ascl2*^BAC^ placentae were not significantly different in weight relative to control placentae at either stage. DelTel7^BAC^ placentae were significantly heavier than the placentae of control littermates at both E14.5 and E18.5. (B) Corresponding fetal wet weights at E14.5 and E18.5. Although *Ascl2*^BAC^ fetuses were slightly heavier than control littermates at E14.5, fetal weight was normalised at E18.5. In contrast, DelTel7^BAC^ fetuses did not differ in weight relative to that of control fetuses at E14.5, but they were substantially growth restricted at E18.5. (C) Fetal:placental (F:P) ratios at E14.5 and E18.5. Placental efficiency was slightly increased for *Ascl2*^BAC^ at E14.5, but was normalised at E18.5. Efficiency of DelTel7^BAC^ placentae was reduced at both E14.5 and E18.5. Numerical data are provided in Table S1. *P<0.05, ***P<0.005.

Two diagnostic characteristics of BWS are fetal overgrowth and placentomegaly at term. Consistent with this, DelTel7^BAC^ placentae (P) were significantly heavier than the placentae of control littermates at both E14.5 (114%; *P*=6.26×10^−10^) and E18.5 (130%; *P*=1.16×10^−14^) (Fig. 2A). However, although there was no difference in fetal (F) weights at E14.5, DelTel7^BAC^ fetuses were significantly lighter than controls at E18.5 (88%; *P*=2.13×10^−9^) (Fig. 2B). Consequently, the F:P ratio, an approximation of placental efficiency (Fowden et al., 2009), was significantly reduced at both E14.5 (89%; *P*=0.0167) and E18.5 (68%; *P*=3.95×10^−22^) (Fig. 2C).

### Progressive loss of the junctional zone in DelTel7^BAC^ placentae

**Fig. 3.**
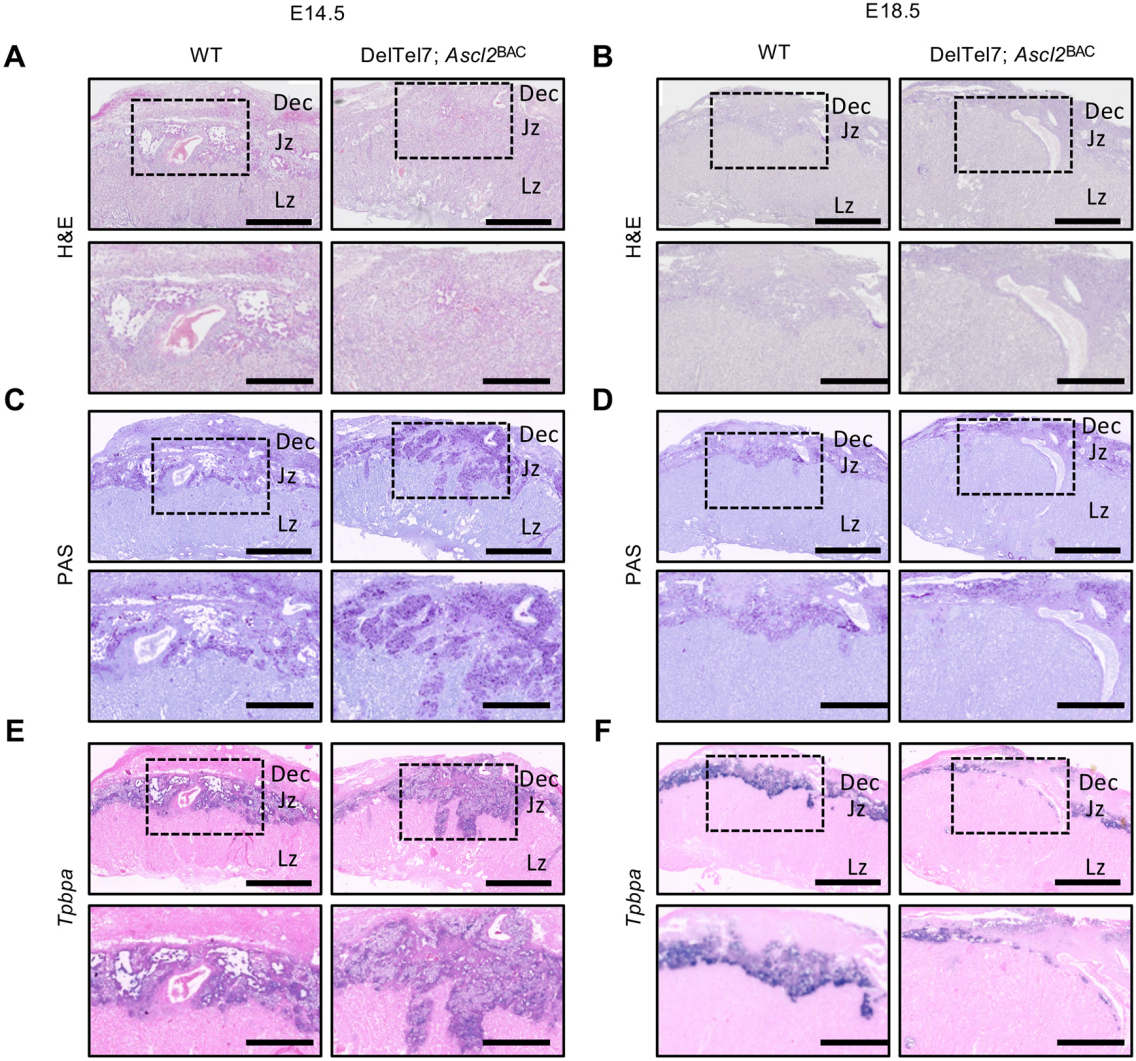
**Disrupted and diminished Jz.** (A,B) H&E staining of midline placental sections of control (left) and DelTel7^BAC^ (right) placentae at E14.5 (A) and E18.5 (B). (C,D) PAS staining of adjacent sections demonstrating mislocalisation of GlyT at E14.5 (C) and diminished glycogen staining at E18.5 (D). (E,F) *In situ* hybridisation with a probe for the Jz marker *Tpbpa*, further demonstrating the Jz mislocalisation defect at E14.5 (E) and diminished Jz at E18.5 (F). Boxed areas are shown at higher magnification in the images below. Scale bars: 1000 μm (upper panels) and 500 μm (lower panels).

**Fig. 4.**
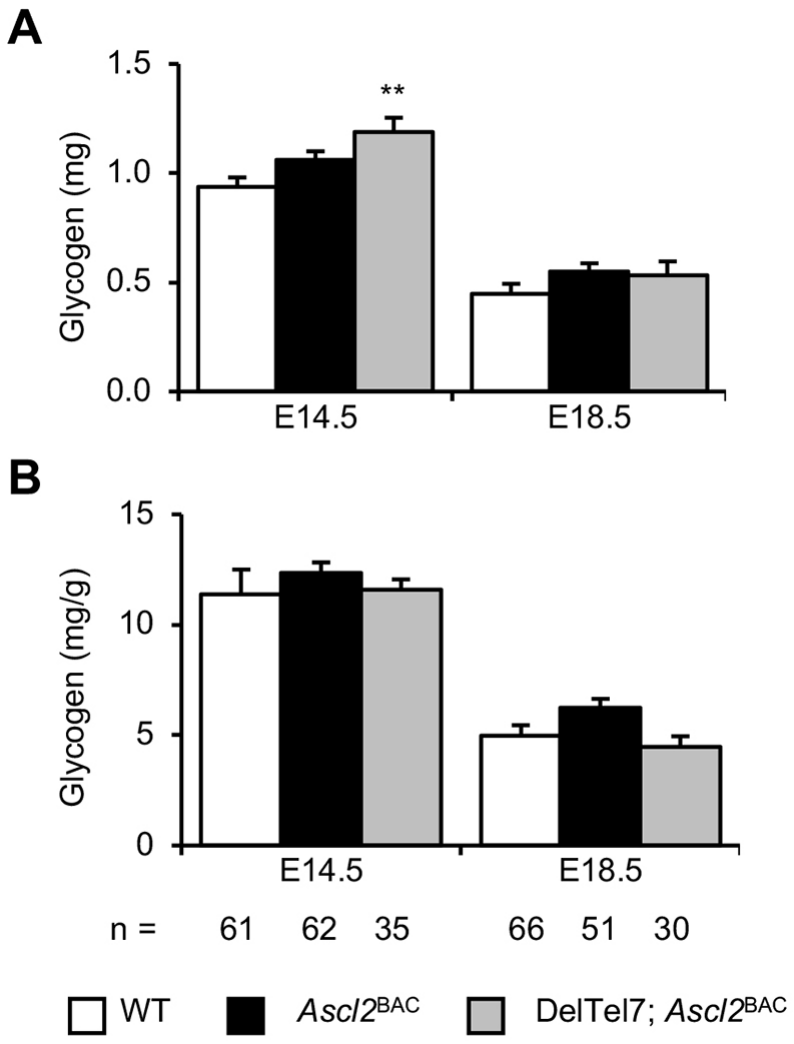
**Transient increase in placental glycogen.** (A) Total placental glycogen of control, *Ascl2*^BAC^ and DelTel7^BAC^ placentae at E14.5 and E18.5. Total placental glycogen stores of DelTel7^BAC^ were ∼25% greater than those of controls at E14.5, but were unaltered at E18.5. (B) When normalised by placental weight, DelTel7^BAC^ placental glycogen stores expressed as milligram of glycogen per gram of placenta were unaltered. Numerical data are provided in Table S2. **P<0.01.

Growth restriction occurred late in gestation in the DelTel7^BAC^ fetuses, and is typically indicative of an extrinsic cause such as placental insufficiency. To further investigate this possibility, we undertook a detailed characterisation of DelTel7^BAC^ placentae. The mature mouse placenta comprises three structurally and functionally distinct layers. The maternal decidua (Dec) forms from uterine cells in response to implantation, the endocrine junctional zone (Jz) is responsible for synthesis and secretion of signalling factors, and the labyrinth zone (Lz) is responsible for nutrient and gas transfer (Watson and Cross, 2005). Haematoxylin and Eosin (H&E) staining of placental midline sections revealed a breakdown in the boundary of the Jz with both the Lz and Dec at E14.5 and E18.5 (Fig. 3A,B). The Jz is primarily composed of glycogen trophoblast cells (GlyT) and the endocrine spongiotrophoblast (SpT). Periodic acid–Schiff (PAS) staining, which stains GlyT, demonstrated a mislocalisation and increased migration of glycogen cells to the decidua at E14.5, although no overt difference in staining was observed at E18.5 (Fig. 3C,D). *In situ* hybridisation with a riboprobe for the Jz marker *Tpbpa* further demonstrated the loss of Jz integrity at E14.5, and revealed a substantial loss of Jz staining at E18.5 (Fig. 3E,F). Biochemical quantitation of placental glycogen stores at E14.5 identified a modest 27% increase in total placental glycogen (*P*=2.61×10^−3^), but this was normalised at E18.5 (Fig. 4A). When adjusted for placental weight, DelTel7^BAC^ placentae did not accumulate significantly more glycogen per gram of placenta at either stage (Fig. 4B).

### Altered gene expression in DelTel7^BAC^ placentae

**Fig. 5.**
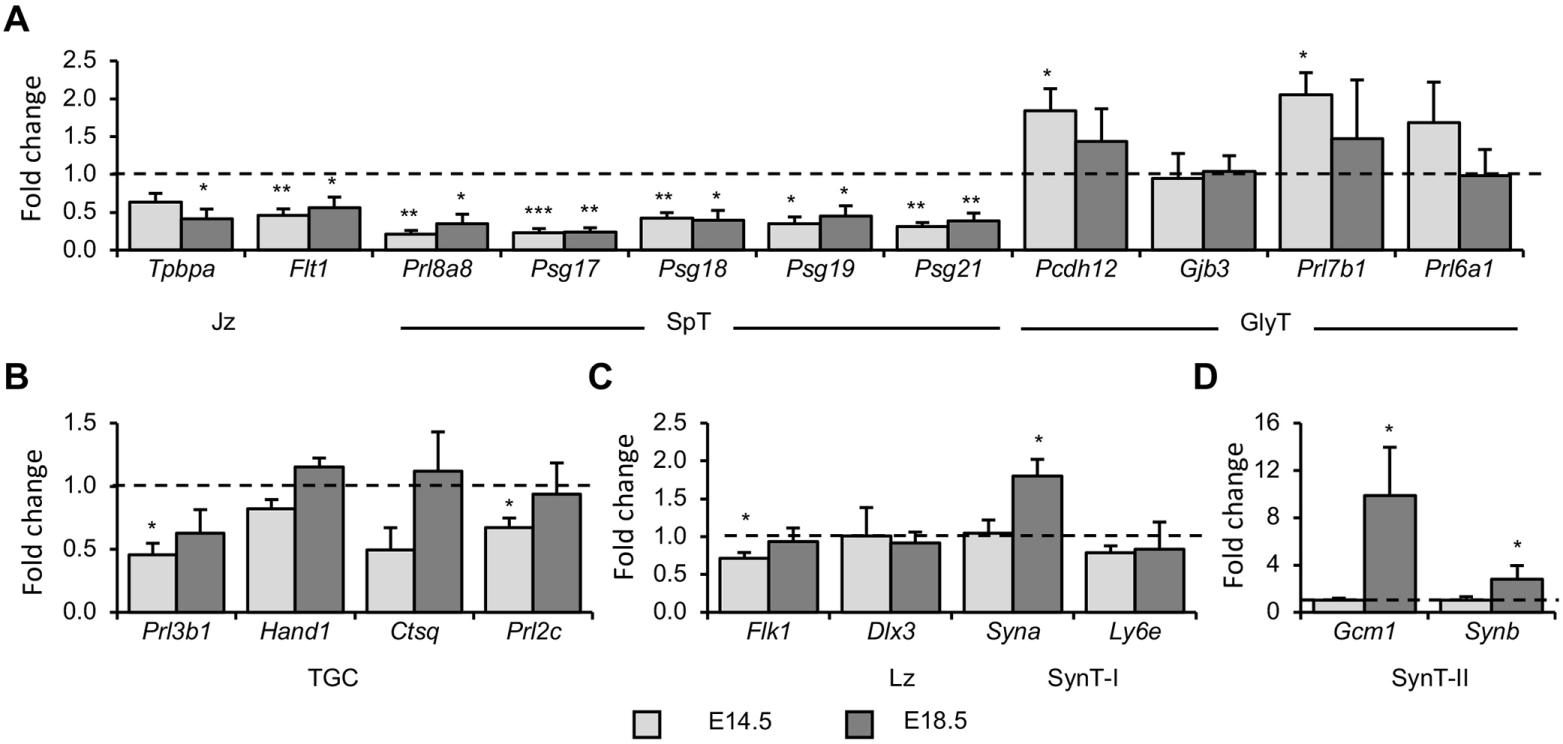
**Relative expression levels of key trophoblast lineage markers.** (A) Expression of Jz and SpT markers was reduced at E14.5 and E18.5, whereas expression of GlyT markers was either slightly elevated or unaltered at these stages. (B) No consistent alteration in expression of TGC markers was observed, with *Prl3b1* and *Prl2c* reduced at E14.5 but not at E18.5, whereas expression of *Hand1* and *Ctsq* was not significantly altered at either stage. (C) *Flk1*, which is predominantly expressed in the fetal vasculature of the labyrinth, was reduced at E14.5 but unaltered at E18.5. Expression of *Dlx3*, which is widely expressed in the labyrinth, was unaltered at both stages. Expression of *Syna* and *Ly6e*, which are both expressed in SynT-I, was unaltered at E14.5. *Syna* was elevated by ∼80% at E18.5, but *Ly6e* remained unaltered at this stage. (D) Expression of the SynT-II markers *Gcm1* and *Synb* was unaltered at E14.5, but dramatically increased at E18.5, with *Gcm1* elevated ∼10-fold and *Synb* elevated ∼4-fold. *n*=4 WT and 4 DelTel^BAC^ from at least two litters. Numerical data are provided in Table S3. *P<0.05, **P<0.01, ***P<0.005.

The mature mouse placenta comprises at least nine distinct trophoblast subtypes (Gasperowicz et al., 2013; John and Hemberger, 2012), each of which is characterised by a unique gene expression profile and spatial organisation. Expression analysis of cell-type-specific gene markers facilitates an assessment of the relative contribution of the various trophoblast lineages to the placenta. Consistent with the gross histological assessment of placental structure (Fig. 3), expression of the Jz marker *Tpbpa* was reduced to 41% of WT levels at E18.5, with a similar trend observed at E14.5 (64%), although without achieving statistical significance (*P*=0.0557) (Fig. 5A). Expression of *Flt1*, which is predominantly expressed in the Jz, was reduced to 46% and 56% of WT levels at E14.5 and E18.5, respectively (Fig. 5A). Expression of genes that are specifically (*Prl8a8*) or predominantly (*Psg17*, *Psg18*, *Psg19*, *Psg21*) expressed in the SpT was substantially diminished at both E14.5 and E18.5, with expression ranging between 21% and 45% of WT levels (Fig. 5A). In contrast, genes expressed either specifically or predominantly in GlyT were either elevated or unaltered in DelTel7^BAC^ placentae. Expression of *Pcdh12*, an early marker of GlyT, was increased by 84% at E14.5, with a similar trend at E18.5, whereas *Gjb3*, a marker of mature glycogen cells (Coan et al., 2006), was unaltered at both E14.5 and E18.5. Expression of *Prl7b1*, a marker of migratory GlyT (Simmons et al., 2008b), was elevated 2-fold at E14.5, consistent with the pattern of PAS staining previously described (Fig. 3B). *Prl6a1*, a marker of nonmigratory GlyT (Simmons et al., 2008b), exhibited a trend for increased expression at E14.5, although without achieving statistical significance, with unaltered expression at E18.5 (Fig. 5A). The reduced expression of Jz markers in DelTel7^BAC^ placentae is consistent with observations in *Cdkn1c*^−/+^ placentae, in which *Tpbpa*, *Prl8a8* and *Flt1* were all downregulated at E15.5 (Tunster et al., 2011).

The boundary between the Jz and Dec is marked by a discontinuous layer of parietal trophoblast giant cells (P-TGC). An additional four trophoblast giant cell (TGC) subtypes have been described, with spiral artery TGCs (SpA-TGCs) lining maternal spiral arteries as they enter the implantation site, canal TGCs (C-TGCs) lining maternal blood canals that traverse the Jz and Lz, and sinusoidal TGCs (S-TGCs) replacing the endothelial lining of maternal blood sinuses in the Lz (Simmons et al., 2007). Finally, the recently described channel TGCs (Ch-TGCs) line venous channels that traverse the Jz carrying blood away from the placenta (Gasperowicz et al., 2013). Expression of *Prl3b1*, which is expressed in SpT in addition to P-, C-, S- and Ch-TGCs, was reduced to 45% of WT levels at E14.5, but was not significantly altered at E18.5 (Fig. 5B). *Hand1*, which is expressed in at least four TGC lineages (Simmons et al., 2007) (expression in Ch-TGCs has not been investigated), was unaltered at both E14.5 and E18.5. Expression of *Ctsq*, which is expressed in S-TGCs and Ch-TGCs (Gasperowicz et al., 2013; Simmons et al., 2007), was expressed at 50% of WT levels at E14.5, although without reaching statistical significance (*P*=0.140), with expression normalised by E18.5 (Fig. 5B). *Prl2c*, which is expressed in SpT in addition to P-, SpA-, C- and Ch-TGCs, was expressed at 67% of WT levels at E14.5 but was unaltered at E18.5 (Fig. 5B). Taken together, these gene expression data are indicative of an early loss of the endocrine SpT population that persists to term, with a transient increase in GlyT at mid-gestation that is normalised by term.

### Elevated expression of syncytiotrophoblast markers

Trophoblast cells in the Lz are arranged in a trilaminar structure, with a layer of S-TGCs replacing maternal endothelial cells, adjacent to which is a bilayer of multinucleated syncytiotrophoblast cells (SynT-I and SynT-II) formed by cell fusion. The endothelial lining of fetal vessels remains intact and lies adjacent to SynT-II (Rossant and Cross, 2001; Simmons and Cross, 2005; Simmons et al., 2008a). Expression of *Flk1* (also known as *Kdr*), which is expressed in fetal endothelium (Hirashima et al., 2003), was reduced to 71% of normal levels at E14.5, but was unaltered at E18.5. Expression of *Dlx3*, which is widely expressed in all Lz trophoblasts (Simmons et al., 2008a), was unaltered at both stages. *Syna* and *Ly6e*, which are expressed predominantly in SynT-I (Hughes et al., 2013; Simmons et al., 2008a), were unaltered at E14.5, with an 80% increase in *Syna* expression at E18.5, whereas *Ly6e* expression remained unaltered at this stage (Fig. 5C). Expression of *Gcm1* and *Synb*, both markers of SynT-II (Simmons et al., 2008a), were unaltered at E14.5, but were elevated ∼10-fold and ∼3-fold at E18.5, respectively (Fig. 5D). DelTel7^BAC^ placentae were therefore characterised by defects in both Jz and Lz.

### Excluding a role for elevated *Ascl2*

In our initial work with *Ascl2*^BAC^, we reported that, in the small intestine, *Ascl2* expression was 2.7-fold higher than endogenous levels (Reed et al., 2012). More recently, we reported that placental *Ascl2* expression exceeds the endogenous levels by 6-fold (Tunster et al., 2016). Thus, a potential shortcoming of this model is the concomitant overexpression of *Ascl2* in the context of loss of expression of the IC2 domain genes. However, we have also demonstrated that the placental defects associated with elevated *Ascl2* were dependent upon *Phlda2*, with *Ascl2* unable to restrict the SpT on a *Phlda2* null background (Tunster et al., 2016). We therefore hypothesised that excess *Ascl2* would have no phenotypic consequence on the context of maternal inheritance of the DelTel7 deletion allele, in which expression of *Phlda2* is ablated. To further explore this hypothesis, we investigated the phenotypic outcomes associated with *Ascl2*^BAC^ combined with loss of function of either *Phlda2* or *Cdkn1c* in isolation.

Transgenic *Ascl2*^BAC^ males were mated with females carrying either a *Phlda2* or *Cdkn1c* loss-of-function allele. Twenty-six litters were generated from crossing the *Phlda2* null line with *Ascl2*^BAC^ males, comprising 187 viable conceptuses, and 20 litters were generated from crossing the *Cdkn1c* null line with *Ascl2*^BAC^ males, comprising 150 viable conceptuses. All genotypes were recovered at the expected frequency at E18.5 (Table 2).

**Table 2.**
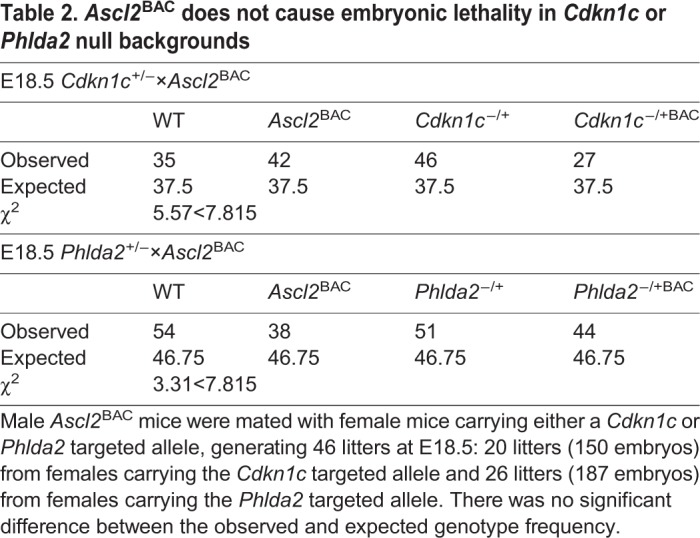
***Ascl2*^BAC^ does not cause embryonic lethality in *Cdkn1c* or *Phlda2* null backgrounds**

**Fig. 6.**
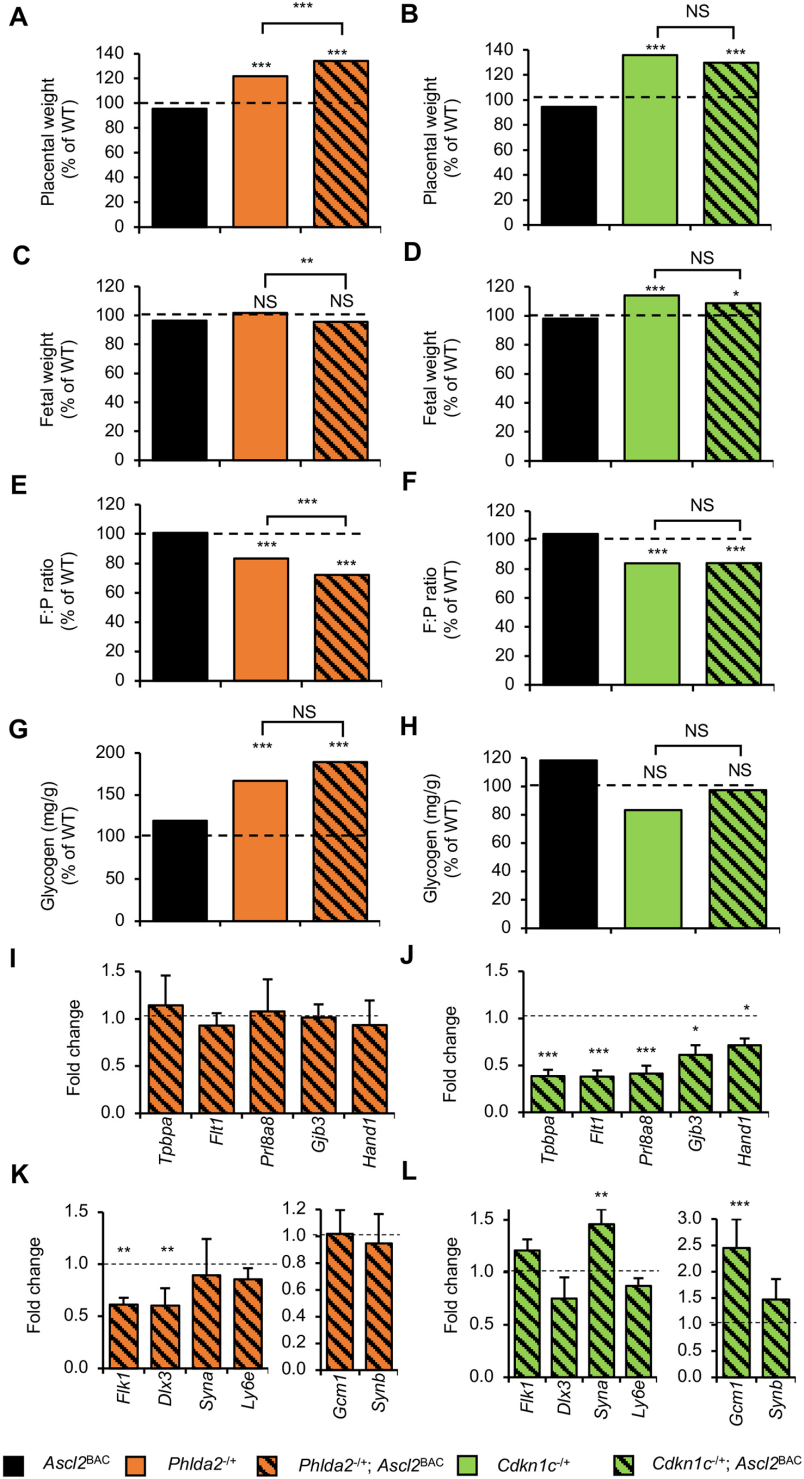
**Excluding a contribution of elevated *Ascl2*.** To exclude a contribution of elevated *Ascl2* in the observed phenotypes we investigated the phenotypic outcomes associated with *Ascl2*^BAC^ combined with loss of function of either *Phlda2* or *Cdkn1c* in isolation at E18.5. (A,B) Placental weights from matings between *Ascl2*^BAC^ males and females carrying either a *Phlda2* (A) or *Cdkn1c* (B) loss-of-function allele. Consistent with previous reports, *Phlda2*^−/+^ and *Cdkn1c*^−/+^ placentae were substantially heavier than the placentae of control littermates. Elevated expression of *Ascl2*^BAC^ did not affect placental overgrowth of *Cdkn1c*^−/+^, with *Phlda2*^−/+BAC^ placentae significantly heavier than those of controls and *Phlda2*^−/+^. (C) The weights of *Phlda2*^−/+^ and *Phlda2*^−/+BAC^ fetuses did not differ from those of control littermates, although *Phlda2*^−/+BAC^ fetuses were slightly lighter than *Phlda2*^−/+^ fetuses. (D) *Cdkn1c*^−/+^ and *Cdkn1c*^−/+BAC^ fetuses were substantially heavier than control littermates, with no significant difference in fetal weight between *Cdkn1c*^−/+^ and *Cdkn1c*^−/+BAC^. (E,F) The substantial placentomegaly, coupled with the absence of any negative effect on fetal growth, resulted in substantially diminished measures of placental efficiency (F:P ratio) for all genotypes except *Ascl2*^BAC^. (G,H) Glycogen content was similarly increased in *Phlda2*^−/+^ and *Phlda2*^−/+BAC^ placentas, but unaltered in *Cdkn1c*^−/+^ and *Cdkn1c*^−/+BAC^ placentas. (I,K) Key lineage markers were largely expressed at wild-type levels in *Phlda2*^−/+BAC^ placentas, with the exception of the Lz markers *Flk1* and *Dlx3*. (J,L) *Cdkn1c*^−/+BAC^ placentas were characterised by diminished expression of Jz markers and increased expression of the syncytiotrophoblast markers *Syna* and *Gcm1*. For A, C and E: WT, *n*=54; *Ascl2*^BAC^, *n*=38; *Phlda2*^−/+^, *n*=51; *Phlda2*^−/+BAC^, *n*=44. For B, D and F: WT, *n*=35; *Ascl2*^BAC^, *n*=42; *Cdkn1c*^−/+^, *n*=46; *Cdkn1c*^−/+BAC^, *n*=27. For G: WT, *n*=47; *Ascl2*^BAC^, *n*=29; *Phlda2*^−/+^, *n*=36; *Phlda2*^−/+BAC^, *n*=32. For H: WT, *n*=40; *Ascl2*^BAC^, *n*=44; *Cdkn1c*^−/+^, *n*=38; *Cdkn1c*^−/+BAC^, *n*=22. For I, J, K and L, *n*=4 per genotype from at least two litters. Numerical data are provided in Table S4. NS, *P*>0.05. *P<0.05, **P<0.01, ***P<0.005.

Consistent with previous reports (Frank et al., 2002; Tunster et al., 2015, 2011), both *Phlda2*^−/+^ (122%; *P*=6.73×10^−12^) and *Cdkn1c*^−/+^ (136%; *P*=1.00×10^−20^) placentae were significantly heavier than the placentae of control littermates. Similarly, both *Phlda2*^−/+BAC^ (134%; *P*=1.00×10^−16^) and *Cdkn1c*^−/+BAC^ (130%; *P*=6.66×10^−16^) placentae were significantly heavier than those of control littermates (Fig. 6A,B). *Phlda2*^−/+BAC^ placentae were heavier than *Phlda2*^−/+^ placentae (110%; *P*=1.82×10^−4^), but there was no difference in placental weight between *Cdkn1c*^−/+^ and *Cdkn1c*^−/+BAC^ (96%; *P*=0.0946) (Fig. 6A,B). Importantly, the fetal weight of *Phlda2*^−/+^ (102%; *P*=0.604) and *Phlda2*^−/+BAC^ (96%; *P*=0.0752) did not differ significantly from that of control littermates, although *Phlda2*^−/+BAC^ fetuses were slightly lighter than *Phlda2*^−/+^ fetuses (94%; *P*=0.00416) (Fig. 6C). We previously reported that *Cdkn1c*^−/+^ embryos on a 129 genetic background were 15% heavier than control littermates at E15.5 and 8% heavier at E18.5, with this slowdown in fetal growth trajectory late in gestation attributable to the associated placental defects (Tunster et al., 2011). On this 129×CD1 genetic background, *Cdkn1c*^−/+^ embryos were 14% heavier than control littermates at E18.5 (*P*=6.21×10^−6^), with *Cdkn1c*^−/+BAC^ 9% heavier than controls (*P*=0.0232), although fetal weight did not differ significantly between *Cdkn1c*^−/+^ and *Cdkn1c*^−/+BAC^ (95%; *P*=0.155) (Fig. 6D). F:P ratios were significantly reduced for all genotypes as a result of substantial placentomegaly (with the exception of *Ascl2*^BAC^) (Fig. 6E,F).

Similarly, *Ascl2*^BAC^ did not overtly affect placental phenotype in the context of loss of function of *Phlda2* or *Cdkn1c*. For instance, the increased placental glycogen associated with loss of *Phlda2* was not influenced by the presence of *Ascl2*^BAC^ (Fig. 6G), and although both *Cdkn1c*^−/+^ and *Cdkn1c*^−/+BAC^ placentae exhibited a trend for reduced placental glycogen this did not achieve statistical significance (Fig. 6H). Key lineage markers were typically expressed at normal levels in *Phlda2*^−/+BAC^ placentae (Fig. 6I,K), whilst the expression profile of *Cdkn1c*^−/+BAC^ placentae was largely consistent with previous reports of *Cdkn1c*^−/+^ placentae (Fig. 6J,L) (Tunster et al., 2011). Importantly, consistent with DelTel7^BAC^ placentae, expression of the SynT-I marker *Syna* and the SynT-II marker *Gcm1* was upregulated in *Cdkn1c*^−/+BAC^ placentae, although upregulation of *Gcm1* was less severe (2.5-fold vs 10-fold, respectively), consistent with the more severe DelTel7^BAC^ phenotype resulting from the combined loss of *Cdkn1c* and *Phlda2*.

Taken together, these data support the conclusion that *Ascl2*^BAC^ does not contribute significantly to fetal growth restriction in the context of loss of function of *Cdkn1c* or *Phlda2* in isolation, with no adverse effect on *Phlda2*^−/+BAC^ fetal growth and *Cdkn1c*^−/+BAC^ fetuses retaining the overgrowth inferred by loss of function of *Cdkn1c*.

## DISCUSSION

This work sought to establish a novel genetic model of sporadic BWS associated with loss of maternally expressed genes in the ICR2 imprinted domain. Loss of expression of the IC2 domain imprinted genes was modelled by maternal inheritance of the DelTel7 truncation allele, with co-inheritance of *Ascl2*^BAC^ to rescue the embryonic lethality caused by loss of *Ascl2* in the mouse. Although the placentomegaly of DelTel7^BAC^ conceptuses was consistent with BWS, we did not recapitulate fetal overgrowth, which was previously considered a defining characteristic of BWS (Brioude et al., 2018; Weksberg et al., 2010). Our findings share some similarity with our previous characterisation of the *Cdkn1c*^−/+^ model of familial BWS. *Cdkn1c*^−/+^ placentae were substantially heavier than those of controls and had a diminished Jz. Although fetal overgrowth was apparent at E15.5 and E18.5, it was absent at birth. The Lz of *Cdkn1c*^−/+^ placentae was characterised by large thrombotic lesions, which we reasoned compromised placental function, leading to the loss of fetal overgrowth during late gestation (Tunster et al., 2011). Similarly, although DelTel7^BAC^ placentae were substantially heavier than control placentae, we did not observe fetal overgrowth at either E14.5 or E18.5. Although we did not observe the large thrombotic lesions of *Cdkn1c*^−/+^ placentae, the substantially elevated expression of the SynT-II markers *Gcm1* and *Synb* is consistent with a widespread disruption of the trilaminar structure of the Lz. We conclude that the larger placenta associated with both BWS models is unable to support fetal overgrowth as a consequence of severe placental defects that compromise normal placental function. Our data are consistent with loss of function of the maternally expressed genes within the IC2 domain having a more profound consequence on fetal growth in mice than in humans, potentially due to the tighter epigenetic regulation of these genes in mice relative to the human locus.

We previously reported that early fetal overgrowth in the *Cdkn1c*^−/+^ model of familial BWS was lost towards term (Tunster et al., 2011). We suggested that this could be due to severe placental defects that impair the ability of *Cdkn1c*^−/+^ fetuses to compete for shared maternal resources. Alternatively, given the role of *Phlda2* in regulating placental glycogen stores (Tunster et al., 2010), thought to be important for fetal growth (Coan et al., 2006), we hypothesised that a combined loss of function of the two genes might be required for fetal overgrowth to manifest (Tunster et al., 2011). Here, we were able to distinguish between these two possibilities using the DelTel model combined with our *Ascl2* transgene, a model that more closely recapitulates sporadic BWS associated with loss of ICR2-regulated genes. The absence of fetal overgrowth after combined loss of *Cdkn1c* and *Phlda2* supports our first scenario, whereby the severe placental defects as a consequence of loss of *Cdkn1c* prevent late fetal overgrowth. Consistent with this, DelTel7^BAC^ placentae were substantially heavier and possessed a diminished Jz, with significantly elevated expression of the SynT-II markers *Gcm1* and *Synb* at E18.5.

In mice, *Cdkn1c* is fully silenced by layers of epigenetic marks including direct DNA methylation (John and Lefebvre, 2011). However, in humans, the locus lacks local DNA methylation on the paternal allele (Diaz-Meyer et al., 2005) and there is ‘leaky’ expression from the paternal *CDKN1C* allele (Diaz-Meyer et al., 2005; Hatada et al., 1996; Matsuoka et al., 1996; Taniguchi et al., 1997). This suggests that late fetal overgrowth observed in BWS might be attributable to this incomplete silencing of the paternal *CDKN1C* allele in humans, which might be sufficient to prevent the marked placental defect.

There are alternative explanations that must be considered, not in the least that the mouse and human placenta differ substantially both structurally and transcriptionally (Carter, 2018; Soncin et al., 2018), which could prevent the accurate modelling of this disorder in mice. The DelTel7 truncation itself might cause fetal growth restriction as it includes haploinsufficiency of ∼20 biallelically expressed genes located at the telomeric end of distal chromosome 7 (Oh et al., 2008). Arguing against this is the absence of any phenotype associated with paternal inheritance of the DelTel7 allele, in which appropriate expression of the IC1 and IC2 domain genes is maintained, while one copy of the nonimprinted telomeric genes is deleted (Oh et al., 2008). A final possibility is that the combination of the *Ascl2* transgene plus DelTel7 impairs fetal growth. Evidence against this is provided by data showing no growth restriction when the transgene was combined with loss of function of *Cdkn1c* in isolation.

In summary, we successfully used an *Ascl2* BAC transgene to rescue embryonic lethality associated with maternal inheritance of a truncation allele of distal chromosome 7, thus creating a novel mechanistic model of sporadic BWS. Although our model recapitulated the placentomegaly associated with BWS, we did not observe fetal overgrowth. Taken together with all the data in this locus in human and mice, we conclude that it might not be possible to accurately model BWS associated with loss of imprinting at IC2 in mice, owing to differences in the epigenetic regulation of the domain between mice and human and/or functional differences between mouse and human placenta.

## MATERIALS AND METHODS

### Mice

All animal studies and breeding were approved by the Universities of Cardiff ethical committee and performed under a UK Home Office project license (R.M.J.). Mice were housed in a conventional unit on a 12-h light–dark cycle with lights coming on at 06:00, with a temperature range of 21±2°C, and with free access to tap water and standard chow. The DelTel7 strain was generated as described previously (Oh et al., 2008) and was a kind gift from Louis Lefebvre (University of British Columbia, Vancouver, Canada). The *Ascl2*^BAC^, *Cdkn1c*^tm1Sje^ and *Phlda2*^loxP^ targeted alleles were generated as described previously (Frank et al., 2002; Reed et al., 2012; Zhang et al., 1997). The DelTel7 strain was maintained on the CD1 background by paternal transmission of the deletion allele. The *Cdkn1c* and *Phlda2* null lines were maintained on the 129S2/SvHsd (129) background by paternal transmission of the targeted allele. The *Ascl2*^BAC^ line was backcrossed by paternal transmission of the transgene to the 129 background for more than eight generations prior to mating with females of the DelTel7 strain. The *Ascl2*^BAC^ line was subsequently backcrossed for more than six generations onto the CD1 background prior to mating with females of the *Cdkn1c* and *Phlda2* null lines on the 129S2/SvHsd (129) background.

### Weighing studies

Embryonic and placental wet weights were taken at the stated time points after a discernible plug. Embryos and placentae were dissected from extraembryonic membranes, immersed in ice-cold fixative, briefly dried and weighed.

### Histological analyses

Placentae were fixed overnight in phosphate-buffered 4% paraformaldehyde, paraffin embedded and cut into 7 µm sections. H&E staining, *in situ* hybridisation and PAS staining for glycogen were performed as described previously (Tunster et al., 2010).

### Gene expression analysis

Quantitative PCR of reverse transcribed RNA was performed on *n*=4 per genotype, with litter-matched controls (*n*=2+2 per litter) as described previously (Tunster et al., 2010).

### Placental glycogen measurement

Glycogen was extracted from whole placenta as described previously, resuspended in 1 ml H_2_O and diluted 1:2, and glycogen concentration was determined using the phenol-sulphuric acid method (Lo et al., 1970).

### Statistical analyses

The χ^2^ test was performed to determine whether the number of conceptuses observed differed from the expected frequency for each genotype. A one-way ANOVA in conjunction with Bonferroni correction was used to compare fetal and placental weights and placental glycogen content between genotypes. Statistical significance for analysis of gene expression was determined using the Student's *t*-test (two-tailed distribution and two-sample unequal variance) (Schmittgen and Livak, 2008).

## Supplementary Material

Supplementary information
