# Supplementary material for: Fetal growth restriction in a genetic model of sporadic Beckwith–Wiedemann syndrome
Source: Dis Model Mech. 2018 Nov 16;11(11):dmm035832. doi: 10.1242/dmm.035832 (PMC6262809; doi:10.1242/dmm.035832)
Supplement: Supplementary information [file dmm-11-035832-s1.pdf]

Table S1: Data for Figure 2

A: Placental weight (mg)		
	**E14.5**	**E18.5**
**WT**	91.4 mg ± 1.6<br>n = 61	90.2 mg ± 1.2<br>n = 66
***Ascl2*^{BAC}**	86.7 mg ± 1.6<br>n = 62	89.3 mg ± 2.2<br>n = 51
**DelTel7^{BAC}**	104.0 mg ± 1.7<br>n = 35	117.5 mg ± 3.4<br>n = 30
**ANOVA**	$F_{2,155}$ = 23.4,<br>$p = 1.27 \times 10^{-9}$	$F_{2,144}$ = 46.2,<br>$p = 3.10 \times 10^{-16}$
**Ratio and Bonferroni corrected *p* value: WT vs *Ascl2*^{BAC}**	94.8%<br>p = 0.0930	99.0%<br>p = 1.000
**Ratio and Bonferroni corrected *p* value: WT vs DelTel7^{BAC}**	113.8%<br>$p = 6.21 \times 10^{-6}$	130.3%<br>$p = 1.16 \times 10^{-14}$
**B: Fetal weight (g)**		
**WT**	0.282 g ± 0.005<br>n = 61	1.405 g ± 0.015<br>n = 66
***Ascl2*^{BAC}**	0.306 g ± 0.006<br>n = 62	1.364 g ± 0.016<br>n = 51
**DelTel7^{BAC}**	0.287 g ± 0.008<br>n = 35	1.231 g ± 0.023<br>n = 30
**ANOVA**	$F_{2,155}$ = 4.5,<br>p = 0.0124	$F_{2,144}$ = 22.0,<br>$p = 4.48 \times 10^{-9}$
**Ratio and Bonferroni corrected *p* value: WT vs *Ascl2*^{BAC}**	108.3%<br>p = 0.0128	97.1%<br>p = 0.206
**Ratio and Bonferroni corrected *p* value: WT vs DelTel7^{BAC}**	101.8%<br>p = 1.000	87.6%<br>$p = 2.13 \times 10^{-9}$
**C: F:P ratio**		
**WT**	3.1 ± 0.07<br>n = 61	15.7 ± 0.24<br>n = 66
***Ascl2*^{BAC}**	3.6 ± 0.09<br>n = 62	15.6 ± 0.29<br>n = 51
**DelTel7^{BAC}**	2.8 ± 0.08<br>n = 35	10.7 ± 0.34<br>n = 30
**ANOVA**	$F_{2,155}$ = 21.2,<br>$p = 7.12 \times 10^{-9}$	$F_{2,144}$ = 76.6,<br>$p = 2.24 \times 10^{-23}$
**Ratio and Bonferroni corrected *p* value: WT vs *Ascl2*^{BAC}**	114.4%<br>$p = 1.76 \times 10^{-4}$	98.9%<br>p = 1.000
**Ratio and Bonferroni corrected *p* value: WT vs DelTel7^{BAC}**	88.6%<br>p = 0.0184	68.0%<br>$p = 4.60 \times 10^{-22}$

Disease Models & Mechanisms • Supplementary information

Table S2: Data for Figure 4

A: Glycogen (mg)		
	**E14.5**	**E18.5**
**WT**	0.94 mg ± 0.039<br>n = 61	0.45 mg ± 0.044<br>n = 60
***Ascl2*^BAC^**	1.06 mg ± 0.052<br>n = 58	0.55 mg ± 0.037<br>n = 49
**DelTel7^{BAC}**	1.19 mg ± 0.048<br>n = 30	0.53 mg ± 0.065<br>n = 27
**ANOVA**	$F_{2,146}$ = 6.1,<br>p = 0.00293	$F_{2,133}$ = 1.5,<br>p = 0.224
**Ratio and Bonferroni corrected *p* value: WT vs *Ascl2*^{BAC}**	113.5%<br>p = 0.122	122.4%<br>p = 0.224
**Ratio and Bonferroni corrected *p* value: WT vs DelTel7^{BAC}**	127.0%<br>p = 0.00261	118.3%<br>p = 0.791
**B: Glycogen (mg/g)**		
**WT**	11.37 mg/g ± 1.13<br>n = 61	4.97 mg/g ± 0.47<br>n = 60
***Ascl2*^{BAC}**	12.34 mg/g ± 0.48<br>n = 58	6.22 mg/g ± 0.42<br>n = 49
**DelTel7^{BAC}**	11.57 mg/g ± 0.49<br>n = 30	4.45 mg/g ± 0.50<br>n = 27
**ANOVA**	$F_{2,146}$ = 0.39,<br>p = 0.679	$F_{2,133}$ = 3.24,<br>p = 0.0422
**Ratio and Bonferroni corrected *p* value: WT vs *Ascl2*^{BAC}**	108.5%<br>p = 1.000	125.1%<br>p = 0.140
**Ratio and Bonferroni corrected *p* value: WT vs DelTel7^{BAC}**	101.7%<br>p = 1.000	89.5%<br>p = 1.000

Disease Models & Mechanisms • Supplementary information

Table S3: Data for Figure 5

**A: Jz markers**			**B: TGC markers**		
	**E14.5**	**E18.5**		**E14.5**	**E18.5**
Tpbpa	0.64 ± 0.11<br>$p = 0.0557$	0.41 ± 0.13<br>$p = 0.0232$	***Prl3b1***	0.45 ± 0.09<br>$p = 0.0261$	0.63 ± 0.19<br>$p = 0.161$
Flt1	0.46 ± 0.08<br>$p = 1.15 \times 10^{-3}$	0.56 ± 0.14<br>$p = 0.0220$	***Hand1***	0.82 ± 0.07<br>$p = 0.0592$	1.15 ± 0.07<br>$p = 0.0637$
Prl8a8	0.21 ± 0.05<br>$p = 6.83 \times 10^{-3}$	0.35 ± 0.13<br>$p = 0.0122$	***Ctsq***	0.50 ± 0.17<br>$p = 0.140$	1.12 ± 0.31<br>$p = 0.715$
Psg17	0.23 ± 0.05<br>$p = 8.64 \times 10^{-5}$	0.24 ± 0.06<br>$p = 0.00366$	***Prl2c***	0.67 ± 0.08<br>$p = 0.0186$	0.93 ± 0.25<br>$p = 0.803$
Psg18	0.42 ± 0.08<br>$p = 4.87 \times 10^{-3}$	0.39 ± 0.13<br>$p = 0.0171$	**C: Lz and SynT-I markers**		
Psg19	0.35 ± 0.09<br>$p = 0.0182$	0.45 ± 0.13<br>$p = 0.0340$	***Flk1***	0.71 ± 0.08<br>$p = 0.0161$	0.93 ± 0.18<br>$p = 0.729$
Psg21	0.31 ± 0.05<br>$p = 0.00191$	0.39 ± 0.10<br>$p = 0.00423$	***Dlx3***	1.01 ± 0.37<br>$p = 0.980$	0.92 ± 0.14<br>$p = 0.612$
Pcdh12	1.84 ± 0.29<br>$p = 0.0138$	1.44 ± 0.43<br>$p = 0.281$	***Syna***	1.05 ± 0.09<br>$p = 0.592$	1.80 ± 0.36<br>$p = 0.0230$
Gjb3	0.95 ± 0.33<br>$p = 0.883$	1.04 ± 0.21<br>$p = 0.843$	***Ly6e***	0.79 ± 0.14<br>$p = 0.209$	0.83 ± 0.11<br>$p = 0.256$
Prl7b1	2.05 ± 0.30<br>$p = 0.0107$	1.48 ± 0.77<br>$p = 0.547$	**B: SynT-II markers**		
Prl6a1	1.69 ± 0.53<br>$p = 0.139$	0.98 ± 0.35<br>$p = 0.956$	***Gcm1***	1.05 ± 0.15<br>$p = 0.767$	9.87 ± 4.08<br>$p = 0.0212$
			Synb	1.03 ± 0.29<br>$p = 0.927$	2.82 ± 1.13<br>$p = 0.0280$

Disease Models & Mechanisms • Supplementary information

Table S4: Data for Figure 6

A: Placental weight (mg)			
	**E18.5**	**Ratio and Bonferroni corrected *p* value vs WT**	**Ratio and Bonferroni corrected *p* value:**
**WT**	87.7 mg ± 1.28<br>n = 54	-	-
***Ascl2*^BAC^** $Ascl2^{BAC}$	83.8 mg ± 1.71<br>n = 38	95.6%<br>p = 0.505	-
$Phlda2^{-/+}$	106.9 mg ± 1.53<br>n = 51	121.9%<br>$p = 6.73 \times 10^{-12}$	**vs** $Phlda2^{-/+}$
$Phlda2^{-/+BAC}$	117.0 mg ± 3.00<br>n = 44	134.2%<br>$p = 1.00 \times 10^{-16}$	110.1%<br>$p = 1.82 \times 10^{-4}$
**ANOVA**	$F_{3,183} = 66.2$, $p = 1.11 \times 10^{-16}$		
**B: Placental weight (mg)**			
	**E18.5**	**Ratio and Bonferroni corrected *p* value vs WT**	**Ratio and Bonferroni corrected *p* value:**
**WT**	84.0 mg ± 1.44<br>n = 35	-	-
$Ascl2^{BAC}$	79.3 mg ± 1.52<br>n = 42	94.4%<br>p = 0.0495	-
$Cdkn1c^{-/+}$	114.1 mg ± 1.58<br>n = 46	135.8%<br>$p = 1.00 \times 10^{-20}$	**vs** $Cdkn1c^{-/+}$
$Cdkn1c^{-/+BAC}$	109.0 mg ± 2.44<br>n = 27	129.7%<br>$p = 6.66 \times 10^{-16}$	95.5%<br>p = 0.0946
**ANOVA**	$F_{3,146} = 111.5$, $p = 1.11 \times 10^{-16}$		
**C: Fetal weight (g)**			
**WT**	1.29 g ± 0.014<br>n = 54	-	-
$Ascl2^{BAC}$	1.24 g ± 0.021<br>n = 38	96.4%<br>p = 0.154	-
$Phlda2^{-/+}$	1.31 g ± 0.015<br>n = 51	101.7%<br>p = 0.604	**vs** $Phlda2^{-/+}$
$Phlda2^{-/+BAC}$	1.22 g ± 0.017<br>n = 44	95.6%<br>p = 0.0752	93.9%<br>p = 0.00416
**ANOVA**	$F_{3,183} = 5.29$, $p = 1.60 \times 10^{-3}$		
**D: Fetal weight (g)**			
**WT**	1.29 g ± 0.023<br>n = 35	-	-
$Ascl2^{BAC}$	1.27 g ± 0.021<br>n = 42	98.0%<br>p = 0.473	-
$Cdkn1c^{-/+}$	1.47 g ± 0.028<br>n = 46	114.0%<br>$p = 6.21 \times 10^{-6}$	**vs** $Cdkn1c^{-/+}$
$Cdkn1c^{-/+BAC}$	1.40 g ± 0.031<br>n = 27	108.6%<br>p = 0.0232	95.3%<br>p = 0.155
**ANOVA**	$F_{3,146} = 15.4$, $p = 9.92 \times 10^{-9}$		

Disease Models & Mechanisms • Supplementary information

**E: F:P ratio**			
**WT**	14.8 ± 0.25<br>n = 54	-	-
***Ascl2*^{BAC}**	14.9 ± 0.30<br>n = 38	100.8%<br>$p = 0.760$	-
***Phlda2*$^{-/+}$**	12.4 ± 0.20<br>n = 51	83.3%<br>$p = 3.20 \times 10^{-11}$	**vs *Phlda2*$^{-/+}$**
***Phlda2*$^{-/+BAC}$**	10.7 ± 0.27<br>n = 44	72.2%<br>$p = 1.00 \times 10^{-26}$	86.6%<br>$p = 1.57 \times 10^{-5}$
**ANOVA**	$F_{3,183} = 62.5$, $p = 1.11 \times 10^{-16}$		
**F: F:P ratio**			
**WT**	15.5 ± 0.35<br>n = 35	-	-
***Ascl2*^{BAC}**	16.2 ± 0.38<br>n = 42	104.3%<br>$p = 0.369$	-
***Cdkn1c*$^{-/+}$**	13.0 ± 0.31<br>n = 46	84.0%<br>$p = 6.59 \times 10^{-6}$	**vs *Cdkn1c*$^{-/+}$**
***Cdkn1c*$^{-/+BAC}$**	13.0 ± 0.42<br>n = 27	84.1%<br>$p = 8.95 \times 10^{-5}$	100.1%<br>$p = 0.984$
**ANOVA**	$F_{3,146} = 21.1$, $p = 2.14 \times 10^{-11}$		
**G: Glycogen (mg/g)**			
**WT**	5.42 ± 0.26<br>n = 47	-	-
***Ascl2*^{BAC}**	6.47 ± 0.44<br>n = 29	119.4%<br>$p = 0.0719$	-
***Phlda2*$^{-/+}$**	9.04 ± 0.36<br>n = 36	166.9%<br>$p = 2.93 \times 10^{-9}$	**vs *Phlda2*$^{-/+}$**
***Phlda2*$^{-/+BAC}$**	10.25 ± 0.63<br>n = 32	189.2%<br>$p = 9.46 \times 10^{-14}$	113.4%<br>$P = 0.266$
**ANOVA**	$F_{3,140} = 30.9$, $p = 2.11 \times 10^{-15}$		
**H: Glycogen (mg/g)**			
**WT**	4.56 ± 0.38<br>n = 40	-	-
***Ascl2*^{BAC}**	5.39 ± 0.37<br>n = 44	118.2%<br>$p = 0.409$	-
***Cdkn1c*$^{-/+}$**	3.80 ± 0.24<br>n = 38	83.2%<br>$p = 0.363$	**vs *Cdkn1c*$^{-/+}$**
***Cdkn1c*$^{-/+BAC}$**	4.44 ± 0.46<br>n = 22	97.3%<br>$p = 0.828$	116.9%<br>$p = 0.543$
**ANOVA**	$F_{3,140} = 3.75$, $p = 0.0125$		
**I & J: Jz Markers**			
	***Phlda2*$^{-/+BAC}$**	***Cdkn1c*$^{-/+BAC}$**	
Tpbpa	1.14 ± 0.31<br>$p = 0.680$	0.38 ± 0.07<br>$p = 0.00316$	
Flt1	0.93 ± 0.13<br>$p = 0.617$	0.38 ± 0.06<br>$p = 1.07 \times 10^{-4}$	
Prl8a8	1.08 ± 0.34<br>$p = 0.821$	0.41 ± 0.08<br>$p = 0.00726$	
Gjb3	1.02 ± 0.14	0.61 ± 0.10	

Disease Models & Mechanisms • Supplementary information

	p = 0.913	p = 0.0299
Hand1	0.93 ± 0.26<br>p = 0.857	0.71 ± 0.07<br>p = 0.0135
**K & L: Lz markers**		
Flk1	0.61 ± 0.06<br>p = 0.0013	1.21 ± 0.11<br>p = 0.0948
Dlx3	0.61 ± 0.16<br>p = 0.0607	0.75 ± 0.20<br>p = 0.282
Syna	0.89 ± 0.35<br>p = 0.789	1.46 ± 0.14<br>p = 0.00600
Ly6e	0.85 ± 0.11<br>p = 0.279	0.87 ± 0.07<br>p = 0.155
Gcm1	1.02 ± 0.18<br>p = 0.924	2.45 ± 0.54<br>p = 0.00300
Synb	0.95 ± 0.22<br>p = 0.819	1.47 ± 0.39<br>p = 0.194
